# Stenting versus shunting in sight-threatening idiopathic intracranial hypertension: genuine equipoise

**DOI:** 10.1136/pn-2025-004728

**Published:** 2025-12-09

**Authors:** Susan P Mollan, Georgios Tsermoulas, Gabriele Berman, Ahmed K Toma, Robertson Fergus, Phil White, Benjamin R Wakerley, Alexandra J Sinclair

**Affiliations:** 1 Metabolism and Systems Science, University of Birmingham, Birmingham, UK; 2 Department of Ophthalmology, Queen’s University, Kingston, Ontario, Canada; 3 Neurosurgery, University Hospitals Birmingham NHS Foundation Trust, Birmingham, Birmingham, UK; 4 Birmingham Neuro-ophthalmology, University Hospitals Birmingham NHS Foundation Trust, Birmingham, UK; 5 Victor Horsley Department of Neurosurgery, National Hospital for Neurology and Neurosurgery, London, UK; 6 Lysholm Department of Neuroradiology, National Hospital for Neurology and Neurosurgery, London, UK; 7 Newcastle Upon Tyne Hospitals NHS Trust, Newcastle upon Tyne, UK; 8 Department of Neurology, University Hospitals Birmingham NHS Foundation Trust, Birmingham, UK

**Keywords:** CSF, CSF DYNAMICS, NEUROOPHTHALMOLOGY, NEUROSURGERY, NEURORADIOLOGY

## Abstract

This opinion piece discusses the challenges of managing a person with sight-threatening papilloedema due to idiopathic intracranial hypertension (IIH). With no available randomised controlled trials, clinicians often choose a locally available surgical intervention. An increasing number of studies have advocated using dural venous sinus stenting in IIH. Big data studies show that shunts have been the mainstay of surgical treatment for IIH, and recent evidence shows improved outcomes and fewer revision surgeries. There remains genuine equipoise in the choice of intervention between shunting and dural venous stenting in IIH. The IIH Intervention Trial funded by the National Institute of Health Research is underway in the UK, the first randomised control trial to evaluate both of these surgical interventions in people with sight-threatening IIH.

 A key therapeutic aim in patients with active idiopathic intracranial hypertension (IIH) is to resolve papilloedema and preserve vision.^1^ In the UK, around 7%–8% of people living with IIH require a procedure, with cerebrospinal fluid (CSF) diversion surgery (shunting) being the most common (6.40%), followed by dural venous sinus stenting (0.99%) and optic nerve sheath fenestration (0.53%).^2^ Although big data studies provide the coded diagnosis and procedure type, less is known about the indication for surgery and the choice of intervention. The IIH consensus guidelines have specifically recommended reserving interventional procedures for patients with impending sight loss, who require rapid reduction of papilloedema, but where maximal medical treatment has failed or is unlikely to help in a necessarily timely fashion.^3^

All of the interventions (CSF shunting, dural venous sinus stenting and optic nerve sheath fenestration) have been shown, to a variable extent, to help improve or stabilise visual function, reduce headache and, indeed, lower intracranial pressure. All have known complications and revision rates.^4^,^9^ The challenge is that studies to date evaluating these procedures have been uncontrolled and so have failed to account for the natural history of spontaneous improvement over time.^10 11^ Existing studies involved heterogeneous cohorts and often relied on variable, non-standardised outcome measures, with frequent use of binary indicators, such as the presence or absence of papilloedema or headache. This leads to a lack of comparable, high-quality data to support confident clinical decision-making.^12^ These studies have also shown high publication bias owing to their case selection, design and analysis, which may have overestimated or underestimated the effectiveness of an intervention; consequently, the ability to carry out meaningful meta-analysis is limited. The River Stent trial, a single-arm, open-label study evaluating a specific intracranial stent to treat symptomatic venous sinus stenosis in IIH, has recently been published.^13^ The primary safety endpoint was the 1-year rate of major adverse events in those who were recruited. These were compared with historical patients with CSF diversion. By comparing their results to an uncontrolled, historically collected CSF diversion dataset, one with a notably high complication rate of 52%, the study may have overstated the efficacy of stenting.^13^ Of note, a recent study using optimised CSF diversion protocols reported a significantly lower revision rate of 11%.^14^ The Surgical Idiopathic Intracranial Hypertension Treatment Trial tried to evaluate ventriculoperitoneal shunting versus optic nerve sheath fenestration versus medical therapy alone. Each arm included standard medical therapy with acetazolamide and a dietary intervention, which would have made it challenging to define which treatment was the most successful. The lack of recruitment led to its termination.^15^ This was probably due to the lack of investigator equipoise, as the trial included a medical treatment-only arm for a defined study population, which in authors’ opinion would have included those deemed to be surgical cases with a visual field mean deviation ranging from −6dB to −27dB. A fundamental issue in any randomised controlled trial is the degree of equipoise between the interventions to be evaluated. This condition requires the investigator and patient to have no clear preference between the two. The loss of equipoise leads to local bias and, as exemplified, failure to recruit patients, resulting in trial closure.

In the absence of clear evidence identifying which procedure is superior in terms of reducing papilloedema or reducing visual morbidity, it is likely that the surgical options available have largely been driven by local expertise. All interventions can resolve papilloedema (figure 1); therefore, planning a head-to-head surgical trial must incorporate reporting of adverse events, revision rates and, in the context of a universal healthcare system, cost-effectiveness. The IIH Intervention Trial funded by the National Institute of Health Research aims to address this critical gap in high-quality evidence by evaluating the effectiveness of shunting and dural venous sinus stenting to guide treatment decisions in the urgent clinical scenario of sight-threatening papilloedema in IIH.^16^ Optic nerve sheath fenestration was not chosen to be evaluated owing to the lack of available surgeons with the expertise within the UK.

**Figure 1:**
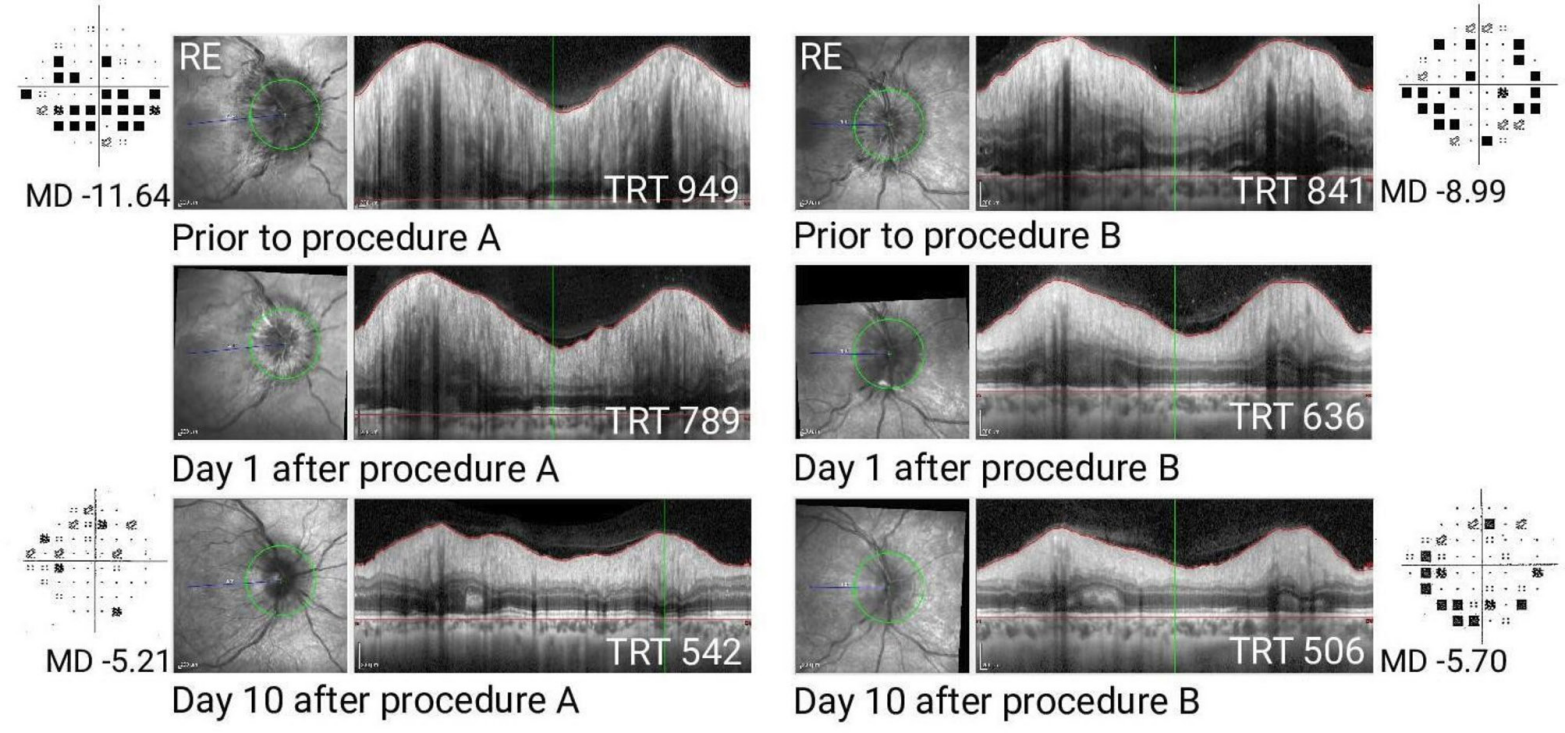
Highlighting that both procedures, CSF shunting and dural venous sinus stenting, can reduce papilloedema and improve visual fields rapidly. Only a randomised control trial can provide evidence on whether one procedure returns better outcomes over the other. Optical coherence tomography (OCT) images of a right optic nerve showing reduction in papilloedema quantified by total retinal thickness and improvement in Humphrey visual field mean deviation following procedure A. Top panel: before the procedure. Middle panel: day 1 following the procedure. Bottom panel: day 10 following the procedure. OCT images of a right optic nerve showing reduction in papilloedema quantified by the total retinal thickness and improvement in Humphrey visual field mean deviation following procedure B. Top panel: before the procedure. Middle panel: day 1 following the procedure. Bottom panel: day 10 following the procedure. LE, left eye; MD, mean deviation; RE, right eye; TRT, total retinal thickness.

An obvious hurdle in trial planning is establishing inclusion criteria in a condition where there are few controlled trials^1017^,^19^ and where disease state classifications are not universally accepted. Two definitions used clinically, although with little validation, are worth mentioning: fulminant IIH and medically refractory IIH.

(1) Fulminant IIH has been defined as those within 4 weeks of their initial IIH diagnosis having escalation of their clinical syndrome requiring surgical intervention.^20^ This term is often applied more flexibly without the time limit because rapid deterioration in IIH can occur at any stage and time-based criteria may delay urgent care.^21^

(2) Many have used medically refractory IIH to describe patients with ongoing headaches, continued pulsatile tinnitus or mild chronic papilloedema. However, most studies do not specify which symptoms are refractory to treatment and whether patients were resistant to weight loss interventions or provide details on the medication classes and dosages used for managing intracranial pressure and headaches. In addition, medically refractory disease would probably be less common if there was rapid access to weight management services, as there is evidence that weight loss correlates with reduction in intracranial pressure and headache burden.^22^ Likewise, the success of modern targeted headache treatments should reduce this group of patients.^23^ In our opinion, those labelled as having medically refractory disease should not currently be offered an intervention where there may be time to engage with successful weight loss interventions and reach optimal medical management with therapeutic doses of medications for appropriate durations.

No single functional or structural measure has been validated to define patients with IIH as an appropriate intervention candidate, nor any biomarkers that can guide prognosis accurately.^24^ It is widely assumed that the Humphrey visual field mean deviation can solely guide clinical decision making, as this is what was the primary outcome in the IIH Treatment Trial.^17 25^ In this trial, they defined eligibility criteria for a medically treated cohort, yet some have inappropriately suggested using the inverse of these criteria to indicate the need for surgical intervention. However, the trial’s visual field eligibility cut-offs were not intended for this purpose. Owing to slow recruitment, this criterion was expanded to include patients out to −7 dB.^17^ Therefore, we need to be mindful of erroneously applying trial criteria, such as mean deviation, which may not be generalisable to the real-world clinic population.^25^ Peripheral visual field constriction is a characteristic of increased intracranial pressure. However, automated visual field tests typically sample only the central 24°–30°, and the peripheral points and points falling at very negative (low) sensitivity thresholds have been shown to be unreliable. Cognitive impairment may also adversely affect visual field mean deviation in IIH.^26^ These factors affect the validity of using the visual field mean deviation as the sole outcome to determine when to proceed to an invasive procedure.^27^

Pragmatically, in the absence of evidence, the IIH Intervention study group have sought to define a surgical candidate in two ways. There has to be moderate or severe and increasing papilloedema in conjunction with associated changes in the mean deviation in a reliably performed visual field assessment.^16^ Of note, moderate papilloedema is defined as a Frisén grade of papilloedema ≥3 as identified by the treating physician,^1^ and increasing papilloedema shown objectively on optical coherence tomography (OCT) images. In an accurate OCT image, where the segmentation has been checked as appropriate, various measures can be used, such as retinal nerve fibre layer thickness, total retinal thickness and measures of optic nerve head volume.^11 24 28^ Those without prior follow-up who have features of sight-threatening papilloedema as evidenced by the presence of a visual field defect and clinically relevant papilloedema, both on clinical examination and on OCT, also meet the inclusion criteria.^16^

Although this article focuses on intervention for those with sight-threatening disease, all healthcare professionals must be mindful of the spectrum of systemic manifestations of IIH that significantly affect patient health and outcomes.^1 29^ These include the metabolic phenotype of truncal adiposity, adipocyte dysfunction, hyperandrogenism, insulin resistance and hyperleptinaemia. There is a two-fold increase in the risk of cardiovascular events compared with those of the same weight, sex, age and fertility and pregnancy complications (gestational diabetes and pre-eclampsia), which typically remain if body weight is not reduced.^29^ The systemic morbidity of IIH is not addressed by lowering the intracranial pressure.

We cannot continue to select patients for interventions based on historical biases, postcode-driven disparities in care and personal physician preferences. There is genuine equipoise for recruiting patients to the IIH Intervention Trial. The results of the IIH Intervention Trial will inform clinical decision-making and change the treatment paradigm for sight-threatening IIH, not only in the UK but far beyond.

**Key points**

- The use of cerebrospinal fluid diversion, dural venous sinus stenting and optic nerve sheath fenestration for idiopathic intracranial hypertension (IIH) has been established for decades; however, no completed randomised controlled trials have compared these interventions, and there remains genuine equipoise regarding which approach is most effective.
- Selecting patients for IIH interventions based on historical biases, postcode-driven disparities in care, personal physician preferences and the absence of randomised trial data is ethically unjustifiable and must be addressed.
- Currently, no single clinical feature can guide clinicians as to when to intervene in sight-threatening IIH; although many believe that the perimetric mean deviation of the visual fields can be used, we caution against this, as this measurement is prone to inaccuracy.
- Optical coherence tomography of the optic nerve head is an established objective measurement to define the severity of papilloedema.
- Neurologists, ophthalmologists, interventional radiologists and neurosurgeons must collaborate particularly in emergency cases where vision is threatened.

## Data Availability

No data are available.
